# Studying hydrogen bonding and dynamics of the acetylate groups of the Special Pair of *Rhodobacter sphaeroides* WT

**DOI:** 10.1038/s41598-019-46903-4

**Published:** 2019-07-19

**Authors:** Daniel Gräsing, Katarzyna M. Dziubińska-Kühn, Stefan Zahn, A. Alia, Jörg Matysik

**Affiliations:** 1 Institut für Analytische Chemie, Universität Leipzig, Linnéstraße 3, D-04103 Leipzig, Germany; 2 Leibniz Institute of Surface Engineering (IOM), Permoserstraße 15, D-04318 Leipzig, Germany; 3 Institut für Medizinische Physik und Biophysik, Universität Leipzig, Härtelstr. 16-18, D-04107 Leipzig, Germany; 4 Leiden Institute of Chemistry, Leiden University, Einsteinweg 55, 2301 RA Leiden, The Netherlands

**Keywords:** Molecular conformation, Solid-state NMR

## Abstract

Although the cofactors in the bacterial reaction centre of *Rhodobacter sphaeroides* wild type (WT) are arranged almost symmetrically in two branches, the light-induced electron transfer occurs selectively in one branch. As origin of this functional symmetry break, a hydrogen bond between the acetyl group of P_L_ in the primary donor and His-L168 has been discussed. In this study, we investigate the existence and rigidity of this hydrogen bond with solid-state photo-CIDNP MAS NMR methods offering information on the local electronic structure due to highly sensitive and selective NMR experiments. On the time scale of the experiment, the hydrogen bond between P_L_ and His-L168 appears to be stable and not to be affected by illumination confirming a structural asymmetry within the Special Pair.

## Introduction

**Figure 1:**
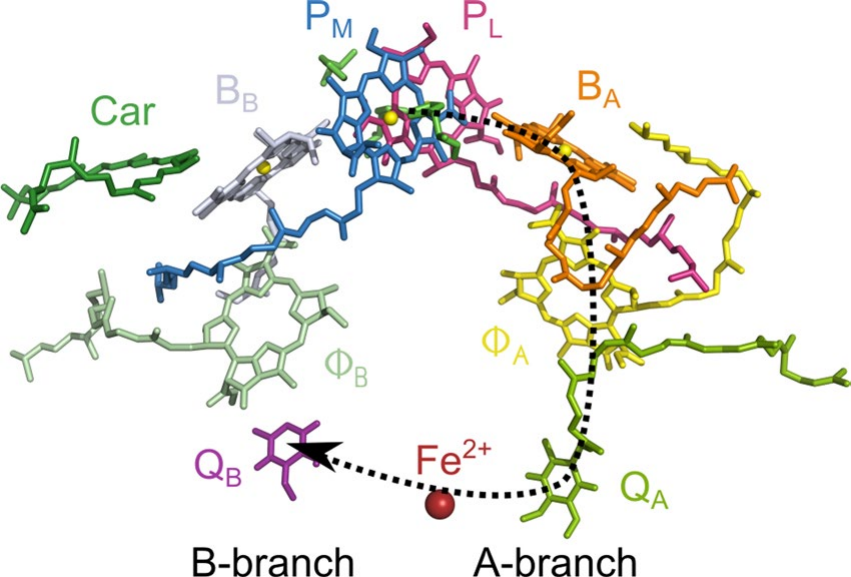
Cofactor arrangement in the RC of *R. sphaeroides* WT: P_M_ and P_L_ ─ bacteriochlorophyll *a* dimer, forming the primary electron donor, the Special Pair P, B_A/B_ ─ accessory bacteriochlorophyll *a*, Φ_A/B_ ─ bacteriopheophytin *a*, Q_A/B_ ─ quinone A and terminal electron accepting quinone B, Fe^2+^ ─ non-heme iron, Car ─ carotenoid. The electron is transferred only via the A-branch (black arrow). For details, see text.

The reaction centre (RC) of the purple bacterium *Rhodobacter (R.) sphaeroides* is a membrane protein in which the primary charge separation, the first step of photosynthesis, is taking place. The availability of the x-ray structure of the RCs of this purple bacterium was a major break-through for the understanding of the early processes in photosynthesis^1^. The cofactors associated with the M- and L-subunits of bacterial RCs are arranged in two nearly symmetric branches spanning the membrane. Each branch consists of two bacteriochlorophylls *a*, a bacteriopheophytin *a* and a quinone. At the end of the branches, a non-heme iron is located (Fig. 1). Close to the B-branch and bound to the M subunit, a carotenoid molecule is present breaking the symmetry of the two branches.

The primary electron-donor P, the so-called Special Pair, is formed by two overlapping bacteriochlorophylls *a*, P_L_ and P_M_. Upon illumination, the Special Pair becomes electronically excited and transfers an electron to the ubiquinone Q_A_ via an accessory bacteriochlorophyll *a* (B_A_) and a bacteriopheophytin *a* (Φ_A_). In the final step, the electron is transferred to Q_B_. Two photocycles coupled with the uptake of two protons reduce Q_B_ to Q_B_H_2_ which diffuses out of the protein into the membrane-based quinone pool.

Although the two branches A and B are nearly symmetric, the electron transfer occurs selectively via the A-branch^2,3^. The directional electron transfer is reflected in the asymmetry of the electronic structure of the Special Pair in the various electronic states. In cation radical state P^•+^ reflecting the HOMO, techniques as EPR, ENDOR and solid-state photo-CIDNP NMR show more unpaired electron spin density on P_L_ than on P_M_^4–7^. The localization of the LUMO mainly on cofactor P_M_, from which the electron transfer occurs, has been explored by the photo-CIDNP MAS NMR analysis of the donor triplet state ^3^P^8^. This asymmetry is already present in the electronic ground-state of the supermolecule P, as demonstrated by differences in chemical shifts^9–13^.

**Figure 2:**
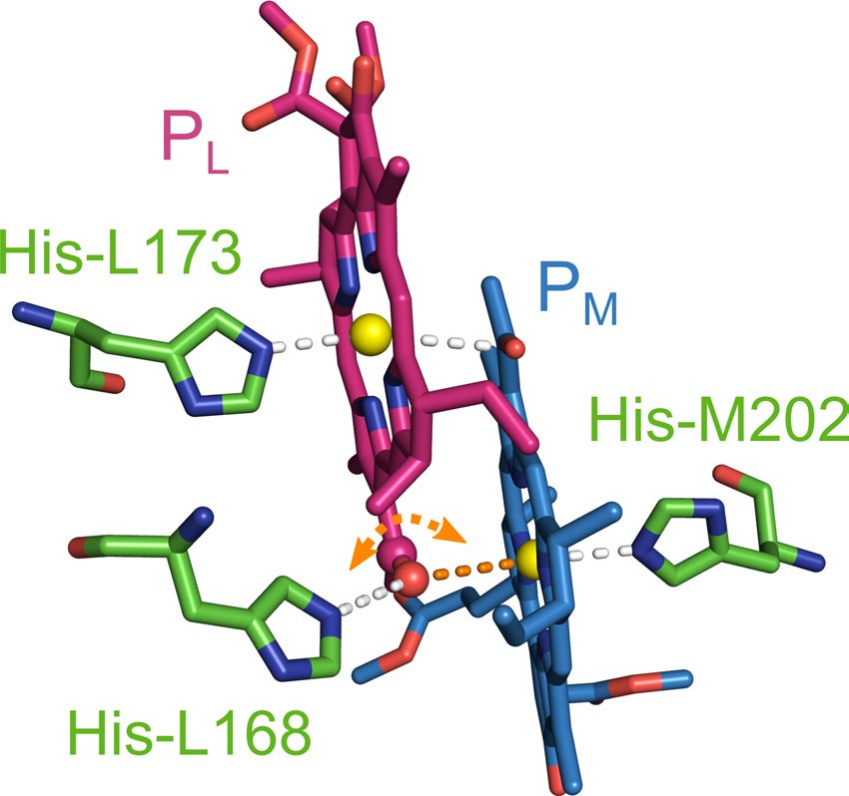
View on the Special Pair with the coordinating histidines. Depending on the protonation state of His-L168, the acetyl group of P_L_ can either be involved in a hydrogen bond (white) or coordinating the magnesium ion of P_M_ (orange). The coordination of the acetyl group might change upon electron transfer (orange arrow), tuning the electronic properties of the Special Pair during its photocycle. The acetyl group of P_M_ is always coordinated to the magnesium ion of P_L_ since no other coordination partner is available.

Mutagenesis as well as theoretical studies established that the orientation and coordination C-3^1^-acetyl groups of P_L_ and P_M_ affect the electronic structure and the redox potential of the special pair^14–16^. As there is no hydrogen bonding partner available, the x-ray structures and Raman spectroscopy data of the acetyl group of P_M_ also show no involvement in any coordination as the Mg-O distance is about 3.3 ± 0.3 Å, while this distance shrinks to 2.4 Å in QM/MM studies “*essentially forming a sixth ligand to the metal”*^17–20^. On the other hand, Raman spectroscopic and QM/MM studies on specifically mutated RC showed that the orientation of the acetyl group of P_L_ depends on the protonation state of His-L168 as it is either involved in a hydrogen bond to His-L168 or, if no hydrogen bond is available, located very close to the magnesium ion of P_M_ (Fig. 2)^18,21–23^. It was therefore suggested that a re-orientation of the acetyl group of P_L_ acts as a valve to block the electron back-transfer upon cleavage of the hydrogen bond to His-L168 and thereby re-tuning of the electronic properties of the Special Pair^16^. The acetyl group might therefore be involved in the reorientation of protein polar groups that lead to electric polarization effects during the radical-pair formation^24–26^. So far, no experimental evidence on the cleavage or the dynamics of the hydrogen bond is known, since no appropriate method with enough sensitivity was available.

**Figure 3:**
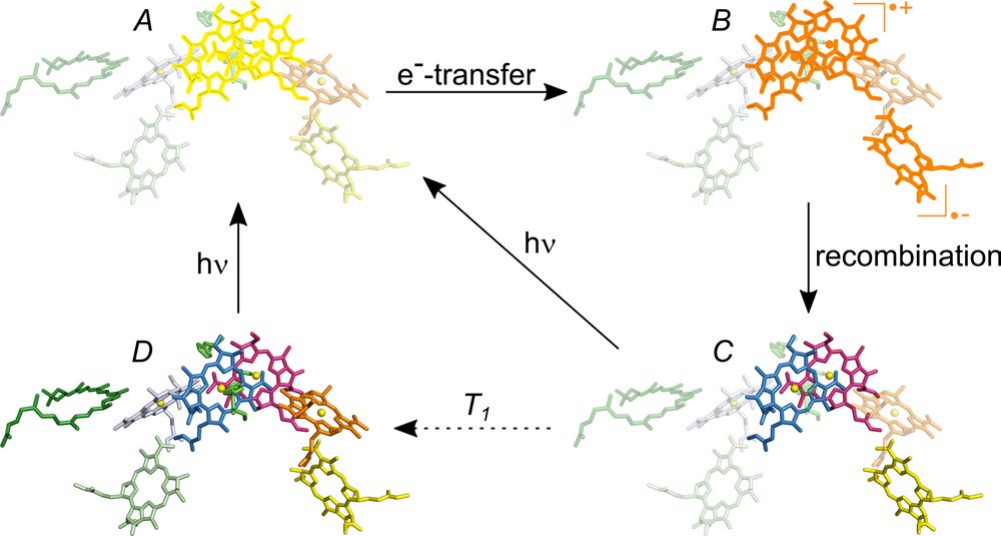
Generation of nuclear hyperpolarisation via the solid-state photo-CIDNP mechanism on the primary donor, the Special Pair P (red and blue), as well as on the primary acceptor Φ_A_ (yellow) in quinone depleted bacterial RC. The phytyl chains are omitted for sake of clarity. After light excitation of P (**A**), an electron is transferred to the Φ_A_ forming a spin correlated radical pair (SCRP) (**B**). In this state, nuclear hyperpolarisation is generated which can be observed in the electronic ground-state after recombination (**C**) (for details, see main text). Hyperpolarisation of P and Φ_A_ is accumulated by a series of photocycles and decays by nuclear *T*_1_ relaxation (**A**/**D**).

Nuclear magnetic resonance (NMR) spectroscopy can be a major technique to probe local dynamics. The lack of sensitivity usually related to this method, can be overcome by the solid-state photo-CIDNP effect allowing to study the photosynthetic cofactors in their native environment^27,28^. The solid-state photo-CIDNP effect induces a non-Boltzmann nuclear spin distribution after a photo-cycle in all natural photosynthetic RC as well as in some flavin proteins^8,29–36^. The enhancement is sufficiently strong to observe particular carbon positions on the cofactors forming the spin-correlated radical pair (SCRP), which is constituted by the donor and the acceptor cofactors, even in entire plants without any further isolation^37^. During the lifetime of the SCRP, multiple coherent mixing mechanisms take place leading to observable nuclear hyperpolarisation in the electronic ground state on the donor and the acceptor molecules. These mechanisms can be explained by level anti-crossings and are termed three-spin mixing (TSM) and differential decay (DD)^38–42^. In case of the quinone-blocked RC of *R. sphaeroides* WT, the Special Pair acts as the donor and the Φ_A_ is the acceptor (Fig. 3).

In this study, we apply photo-CIDNP MAS NMR experiments to investigate the hydrogen-bond interaction between the acetyl group of P_L_ and His-L168 by measuring the chemical shift anisotropy (CSA) of C-3^1^ of the acetyl group and comparing it to DFT calculations to test whether the assumed cleavage of the hydrogen bond between P_L_ and His-L168 can be experimentally verified.

## Methods

### Sample preparation

Cultures of *Rb. sphaeroides* WT were grown anaerobically in the presence of 1.0 mM [3-^13^C]-δ-aminolevulinic acid • HCl (3-ALA) (Buchem B.V., Apeldoorn, The Netherlands) for selective ^13^C isotope labelling of BChl a and BPhe as described before [9]. The RCs were isolated and the quinones were removed as described earlier [12]. Approximately 10 mg of RC protein complex embedded in LDAO micelles was used for the NMR experiment.

### Solid-state photo-CIDNP MAS NMR

All NMR experiments have been performed with a double-resonance MAS probe at a Bruker AVANCE III spectrometer (Bruker-Biospin, Karlsruhe, Germany) operating at a proton Larmor frequency of 400.15 MHz. The probe was equipped with a light fiber to illuminate the sample during the measurement as described in ref.^27^. As illumination source, a 488-nm continuous-wave laser (Genesis MX488–1000 STM OPS-Laser-Diode System, Coherent Europe B.V., The Netherlands) operating at 1 W was used. The sample was packed in a clear 4-mm sapphire rotor and frozen in the dark at a slow spinning frequency of 800 Hz to ensure a homogenous sample distribution^43^.

If not stated differently, NMR experiments were performed at a spinning frequency of 8 kHz with a recycle delay of 4 s and a temperature of 247 K. The $\pi /2$ ^13^C pulses were applied at radio-frequency (rf) field strength of 72 kHz, while the rf field strength of the heteronuclear SW_f_-TPPM decoupling was set to 100 kHz^44^. For the 1D experiment, 1024 scans were recorded with an acquisition time of 20 ms. The spectral width was set to 30 kHz, with the offset placed in the centre of the spectrum, if not stated otherwise. For the 2D INADEQUATE experiments, the SR26 sequence with an rf field strength of 52 kHz was applied^45^. One full SR26 cycle was used for DQ excitation and reconversion each, resulting in a total mixing time of 4 ms. To ensure a large spectral width of 46 kHz, STiC phase shifts were used^46^. The carrier was placed at 100 ppm. A total of 640 scans were averaged per each of the 100 t_1_ increments collected. The t_2_ acquisition time was set to 20 ms. Heteronuclear SW_f_-TPPM decoupling was used during the t_1_ and t_2_ acquisition^44^. The SUPER experiment was performed at a spinning frequency of 6 kHz leading to a rf field of 72.72 kHz for CSA recoupling. 192 scans were averaged during each of the four γ-integral points used, leading to a total number of scans of 768. A total of 32 t_1_-increments were recorded. The spectral width was set to 32258.1 Hz taking into account the scaling factor of 0.155^47^. The carrier was placed at 192 ppm. The z-filter for the γ-integral was set to 100 µs. Heteronuclear SW_f_-TPPM decoupling was used during the t_1_ and t_2_ acquisition^44^. Frequency discrimination in all 2D experiments was achieved using the States-TPPI method^48^.

The simulation of the CSA line shapes were carried out with SIMPSON^49^. The script can be found in the supplementary information.

### DFT calculations

Geometry optimization calculations were based on the crystal structure of Camara-Artigas *et al*. (PDB: 1M3X) and were carried out with the program ORCA 4.0.1.2^50,51^. The resolution of identity approximation in combination with the corresponding auxiliary basis set was employed to speed up the calculation based on the BLYP functional in combination with a def2-SVP basis set^52–54^. The empirical dispersion correction of Grimme 3^rd^ version (with Becke/Johnson) was employed to consider dispersion interactions^55,56^. The protein environment was considered by the conductor-like polarisable continuum model (CPCM) for which a dielectric constant of ε = 4 was selected^57^. The two investigated structural models are shown in the Supplementary Figs S1 and S2. Both models differ in the protonation pattern at His-L168 where solely model A forms a hydrogen bond between His-L168 and P_L_, see Fig. S1. The chemical shifts were calculated with the BLYP functional in ADF 2017 using good numerical quality and no frozen core. The empirical dispersion correction of Grimme 3^rd^ version (with Becke/Johnson) was employed to consider dispersion interactions. For protons a single-zeta basis set without polarization was applied. For the carbon atoms a double-zeta singly polarized Slater-type basis set (DZP) was used. Application of a triple-zeta singly polarized Slater-type basis set (TZP) lowered the obtained agreement in isotropic chemical shifts.

## Results and Discussion

**Figure 4:**
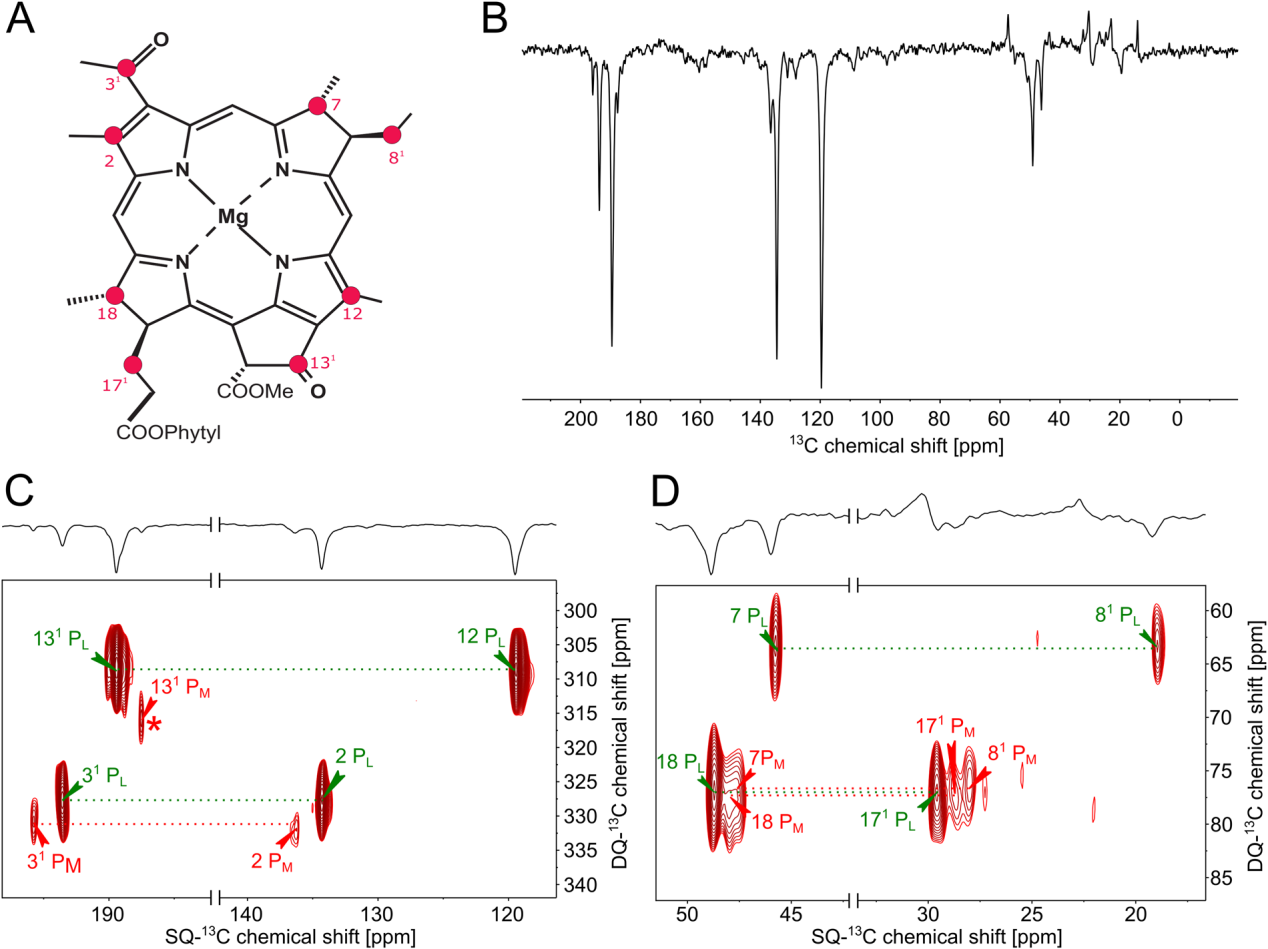
(**A**) Labelling pattern in bacteriochlorophyll *a* achieved by feeding 3-δ-aminolevulinic acid (3-ALA). The atom numeration is according to IUPAC. (**B**) 1D ^13^C spectra of 3-ALA labeled RC of *R. sphaeroides* WT. (**C**,**D**) Detailed views on the low (C) and high (D) field regions of the INADEQUATE spectrum of 3-ALA labeled RC of *R. sphaeroides* WT. The double-quantum peak of C-12 P_M_ correlated to C-13^1^ of P_M_ (marked with an asterisk) was in the range of noise.

So far, the assignment of the resonances of the cofactor signals has been performed by comparison of the resonances with the relevant chlorophyll in solution state or by homonuclear DARR or RFDR experiments^9,10,12,58–61^. Comparison to model molecules can lead to wrong assignments due to the drastic effect of the protein matrix on the electronic structure of the Special Pair. Two-dimensional homonuclear experiments on samples with several tetrapyrrole macrocycles might struggle from signal overlap. We therefore apply INADEQUATE experiments to unambiguously assign the resonances as it has already been performed on the 5-ALA labelling pattern^62^. Since the distances are significantly larger in the 3-ALA labelling pattern (Fig. 4A), we used the SR26 sequences which recouples weak dipolar interactions efficiently^45^.

Figure 4B shows the 1D spectrum as well as a detailed view on the 2D INADEQUATE spectrum (Fig. 4C,D). As can be seen, a clear correlation between neighbouring labeled carbons up to two bonds apart can be established. This connectivity, the fact that P_L_ carries more electron density leading to more shielding, as well as the already known assignments from the DARR spectra allow to assign all resonances unambiguously as shown in Table 1. In course of this, due to the observed correlation signal with C-7 of P_L_ at 64 ppm, we assign the resonance at 19 ppm to C-8^1^ of P_L_ which has been erroneously denoted as C-7^1^ in ref.^12^. We do not observe the resonances of C-8^1^ of P_L_ at 32.1 ppm, C-12 of P_M_ at 128.8 ppm and C-18 of P_M_ at 50.9 ppm as it was reported earlier^12^. This might be due to the strong field dependence of the solid-state photo CIDNP effect and the different magnetic fields used for both experiments^63,64^. Nevertheless, the SR26 sequence shows a good performance and allows for recoupling over about 2.6 Å (i.e., two bonds) making it suitable for even sparsely labeled samples as they are used in photo-CIDNP MAS NMR.

**Table 1:** Experimentally determined isotropic ^13^C chemical shifts of the Special Pair in the 3-ALA ^13^C labeled bacterial RCs of *R. sphaeroides* WT.

Position	P_L_	P_M_
2	134.9 ppm	136.9 ppm
3^1^	194.1 ppm	196.3 ppm
7	46.5 ppm	—
8^1^	19.7 ppm	28.9 ppm
12	120.0 ppm	—
13^1^	188.0 ppm	190 ppm
17^1^	30.1 ppm	29.2 ppm
18	49.39 ppm	—

**Figure 5:**
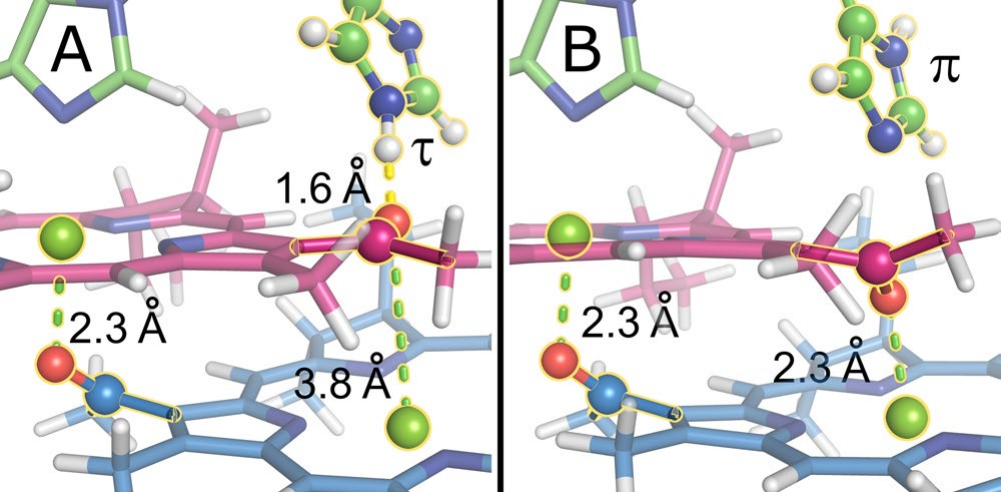
Detailed view on the acetyl group of P_M_ (blue) and P_L_ (pink) in the two DFT models. On the left-hand side (model **A**), His-L168 is protonated in the τ-position forming a hydrogen bond of 1.6 Å to the acetyl group of P_L_ suggesting a moderate hydrogen bonding interaction. The acetyl group of P_M_ does not have a hydrogen bonding partner and is therefore coordinated to the magnesium ion of P_L_. On the right-hand side (model **B**), His-L168 is protonated in the π-position. Lacking a partner for hydrogen bonding, the acetyl group of P_L_ coordinates to the magnesium ion of P_M_. The acetyl group of P_M_ is always coordinated to the magnesium ion of P_L_. The extended presentations of the two models are shown in the Supplementary Information (Supplementary Figs S1 and S2).

Hence, the two C-3^1^-acetyl carbons of P_M_ and P_L_ have different isotropic chemical shifts, occurring at 194.5 (P_L_) and 196.3 ppm (P_M_), pointing towards a different chemical environment as, for example, that the acetyl group of P_L_ has hydrogen bond interaction with His-L168, while the acetyl group of P_M_ coordinates the magnesium of P_L_. To obtain further insight into the chemical environment, we investigated two different DFT models in which His-L168 is protonated at either the $\tau$ or the $\pi$ position (Fig. 5). Depending on the protonation state of His-L168, the acetyl group of P_L_ is either involved in a hydrogen bond to His-L168 (model A) or it coordinates to the magnesium ion of P_M_ (model B).

To verify the existence of the hydrogen bond and to explore possible dynamics, we measured the chemical shift anisotropy (CSA) pattern of both groups via the SUPER technique^47^. In highly enriched samples, SUPER reintroduces homonuclear dipolar interactions which in conjunction with J-coupling leads to broadening of the CSA patterns at slow spinning speeds^47,65^. In our case, moderately fast spinning, only very few labels, weak dipolar interactions (~390 Hz) and the absence of J-couplings should not lead to significant broadening of the CSA pattern as it was verified by SIMPSON simulations (Supplementary Figure S4).

**Figure 6:**
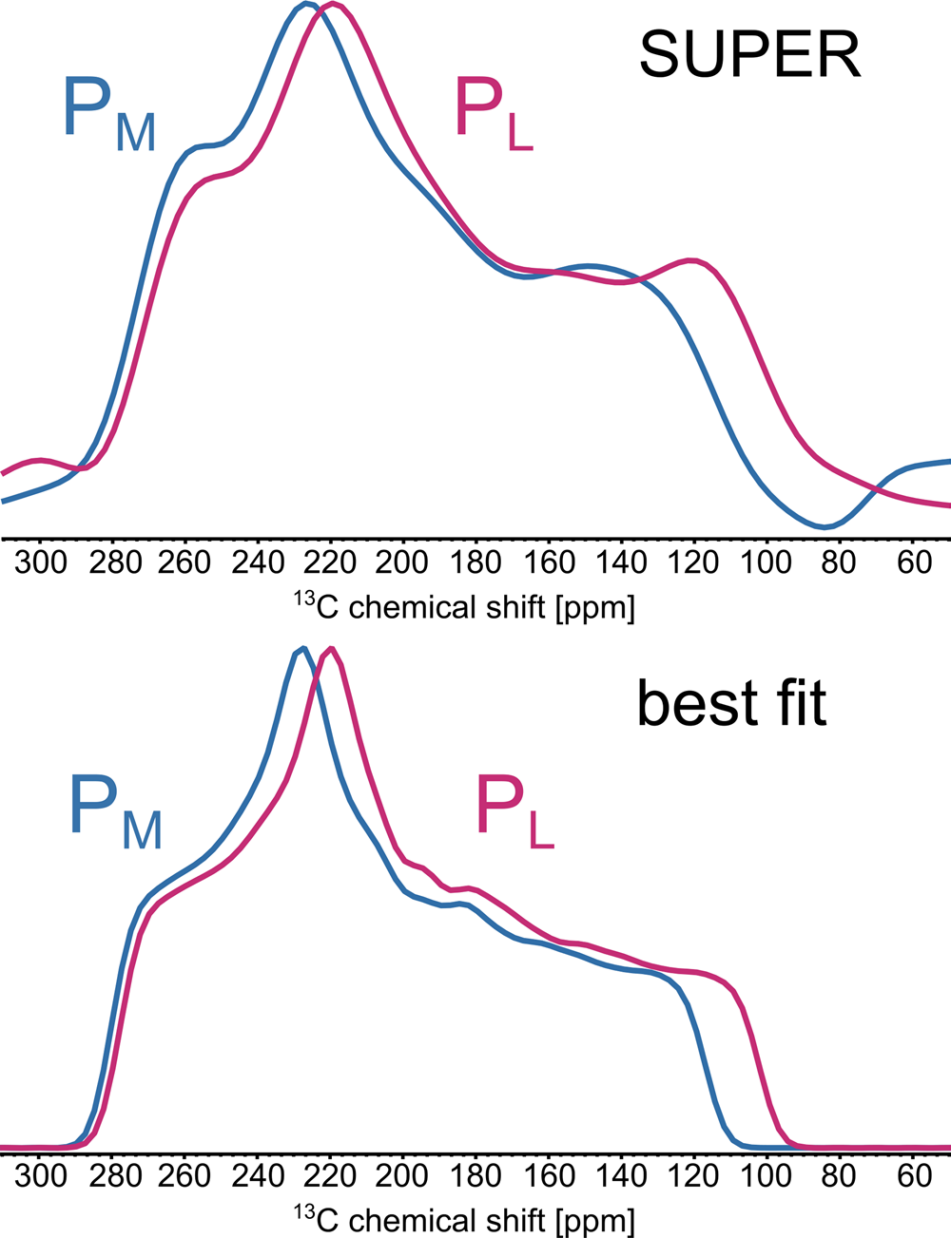
Cross section from the experimental 2D SUPER spectra (top) and the best fit (bottom) patterns of the C-3^1^-carbon in the acetyl groups of P_M_ and P_L_. The principal values of the best fit are summarized in Tab. 2 in the “Experiment P_L_” and “Experiment P_M_” column.

Figure 6 shows the experimental powder patterns of C-3^1^ in the acetyl groups of P_L_ and P_M_ as well as their simulations which matched the experimental data best. Table 2 shows the experimentally obtained isotropic and anisotropic chemical shift values of P_L_ and P_M_ compared to the theoretically obtained isotropic and anisotropic chemical shift values obtained from the DFT calculations where His-L168 is protonated either in τ- or π-position (model A or B). The difference in the principal values of the CSA (δ_11_-δ_33_) of the two molecules show an unequal coordination mode of the two acetyl groups suggesting a hydrogen bond between the acetyl group of P_L_ and His-L168.

**Table 2:** Experimental (best fit from Fig. 2) and calculated isotropic chemical shift ${\delta }_{iso}$, reduced anisotropy ${\delta }_{aniso}$ and principal values *δ*_11_ − *δ*_33_ of the CSA tensor according to IUPAC of the acetyl groups of the two special pair molecules P_L_ and P_M_.

	Experiment P_L_	DFT P_L_	DFT P_L_	Experiment P_M_	DFT P_M_	DFT P_M_
Model	—	A	B	—	A	B
${\delta }_{iso}$ [ppm]	194.1	195.3	204.6	196.3	201.9	200.8
${\delta }_{aniso}$ [ppm]	−120 ± 2	−107	−120	−112 ± 2	−114	−114
η [−]	0.60 ± 0.04	0.24	0.09	0.58 ± 0.04	0.09	0.16
${\delta }_{11}$ [ppm]	290	262	270	285	269	267
${\delta }_{22}$ [ppm]	218	236	259	220	249	249
${\delta }_{33}$ [ppm]	74	88	85	85	88	87

The observed experimental anisotropy values ${\delta }_{aniso}$ of both powder patterns ($12\,\mathrm{kHz}\,\cong 120\,\mathrm{ppm}$ (P_L_) and $11.3\,\mathrm{kHz}\,\cong 112\,\mathrm{ppm}$ (P_M_)) are larger than the anisotropy values of the carboxylate group in glycine (${\delta }_{aniso}\cong 7.5\,{\rm{kHz}}$) providing strong evidence for the high rigidity of the system^47^. Motions with a correlation time ${{\rm{\tau }}}_{{\rm{c}}}\ll 85\,\mu s\,\,$ would lead to an averaging of the CSA, which is in the time scale of multiple photocycles in RC of *R. sphaeroides* WT^66^. Since the sample is under continuous illumination and therefore passes multiple photocycles during each scan, the size of the anisotropy implies that the acetyl group, if there is any structural change related to this group, does not remain changed on the timescale of nanoseconds or longer. If the acetyl group of P_L_ would be changing its orientation, the movement in both directions need to be on a ps time scale that is not observable with this experiment.

The calculated isotropic chemical shifts of C-3^1^ of P_L_ show a reasonable agreement for model A. If His-L168 is protonated in the π-position (model B), the calculated isotropic chemical shift is off by about 10 ppm as C-3^1^ of P_L_ is coordinated to the magnesium ion of P_M_. The isotropic chemical shift as well as the principal values of the chemical shift anisotropy of C-3^1^ of P_M_ are independent of the protonation state of His-L168. We note, however, that the calculated values are off by about 5 ppm which is within the expected error of the employed approach^67^. Unfortunately, we are limited to GGA calculations due to the size of the system. Since the principal CSA values are caused by the electronic environment of the observed nucleus, the differences in the principal CSA values might therefore also be caused by differences in geometry of the model compared to the experimental case which is assumed to be close to the crystal structure. In this case, the high accuracy of the NMR data might be used to recalculate the orientation of the acetyl group and therefore for refinement of the arrangement of the cofactor in the protein pocket.

The findings are also in agreement with the observations of Li and Hong stating that the π-tautomer of histidine is only formed as an anionic tautomer at high pH and is metastable in the presence of water suggesting a short lifetime^68^. Hence, a stabilization of the π-tautomer of His-L168 can only be achieved by further metal-ion coordination or H-bonding, which is implausible in this case^68,69^. Therefore, we assume that His-L168 is protonated in the τ-position.

## Conclusion

We used the selectivity and the strong enhancement of solid-state photo-CIDNP MAS NMR to identify and probe the dynamics of the two acetyl groups in the Special Pair of *R. sphaeroides* WT by measuring the isotropic chemical shifts and principal values of their CSA tensors. In conjunction with DFT calculations, we showed that a rigid hydrogen bond between His-L168 and the acetyl group of P_L_ is present. The high values of the reduced anisotropy of the CSA of both acetyl groups imply that they are not changing their orientation on the time scale of ns to µs. This suggests that if the acetyl-group of P_L_ is flipping to act as a valve preventing fast charge-recombination, the flip has to happen on the ps time scale after the light-induced electron transfer.

## Supplementary information

SI
