# Supplementary material for: Studying hydrogen bonding and dynamics of the acetylate groups of the Special Pair of Rhodobacter sphaeroides WT
Source: Sci Rep. 2019 Jul 19;9:10528. doi: 10.1038/s41598-019-46903-4 (PMC6642110; doi:10.1038/s41598-019-46903-4)
Supplement: Supplementary file 1 — SI [file 41598_2019_46903_MOESM1_ESM.pdf]

Supplementary information

Studying hydrogen bonding and dynamics of the acetylate groups of the Special Pair of *Rhodobacter sphaeroides* WT

Daniel Gräsing[†], Katarzyna M. Dziubińska-Kühn[†], Stefan Zahn[‡], A. Alia[□,□] and Jörg Matysik[†]*

[†] Institut für Analytische Chemie, Universität Leipzig, Linnéstraße 3, D-04103 Leipzig, Germany

[□] Leiden Institute of Chemistry, Leiden University, Einsteinweg 55, 2301 RA Leiden, the Netherlands

[□] Institut für Medizinische Physik und Biophysik, Universität Leipzig, Härtelstr. 16-18, D-04107 Leipzig, Germany

[‡] Leibniz Institute of Surface Engineering (IOM), Permoserstraße 15, D-04318 Leipzig, Germany

Keywords: solid-state photo-CIDNP, bacterial reaction center, chemical shift anisotropy, INADEQUATE, MAS NMR

Corresponding Author

* E-mail address: joerg.matysik@uni-leipzig.de (J. Matysik)

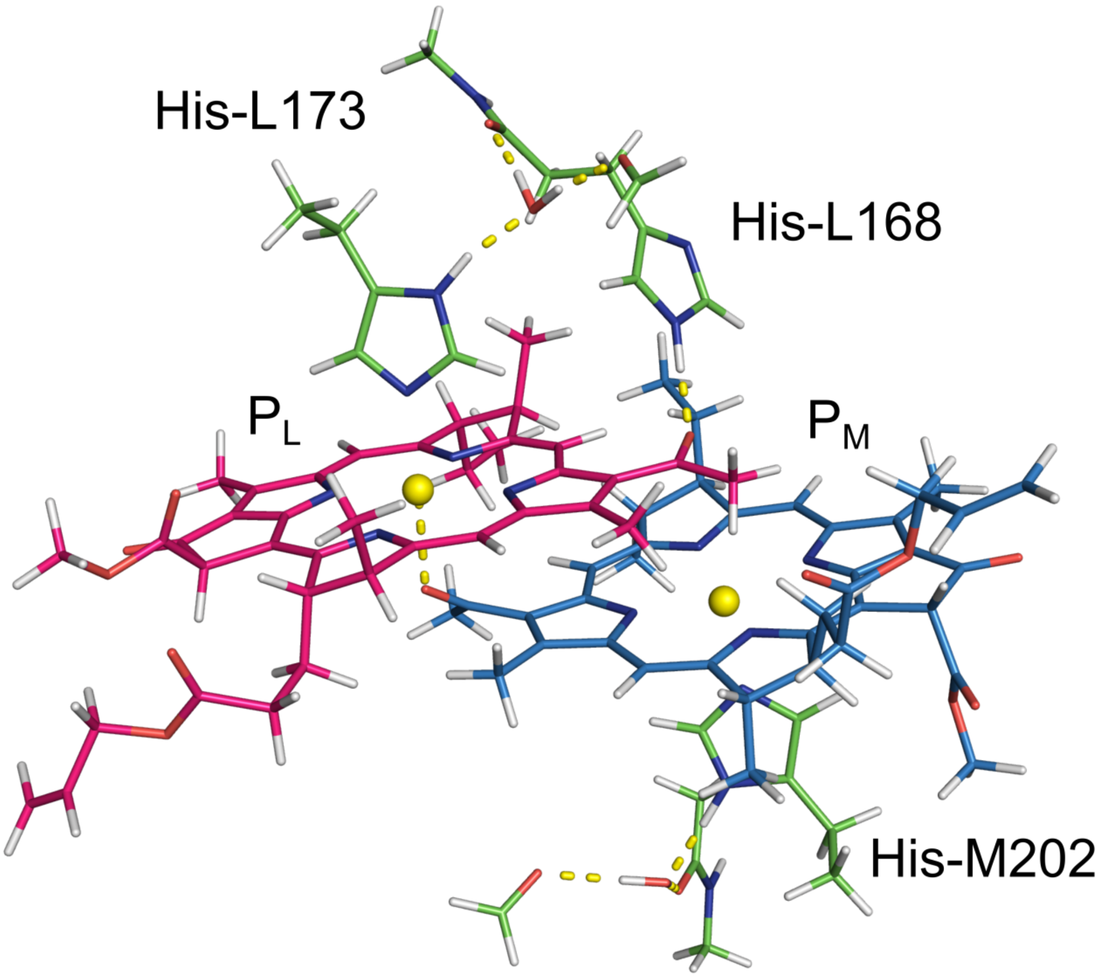

Fig. S1. Full view of the investigated model system where His-L168 is protonated in the τ-position (model A).

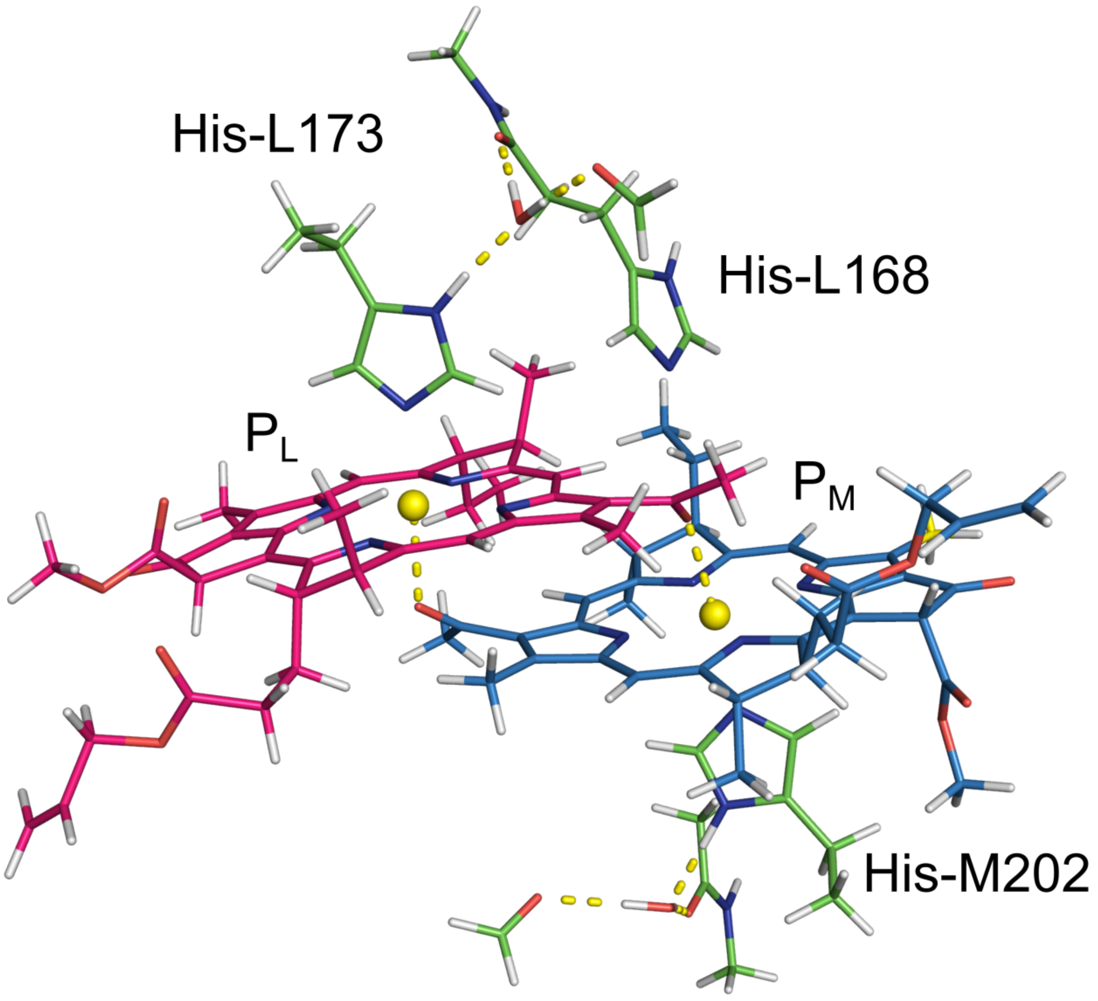

Fig. S2. Full view of the investigated model system where His-L168 is protonated in the π-position (model B).

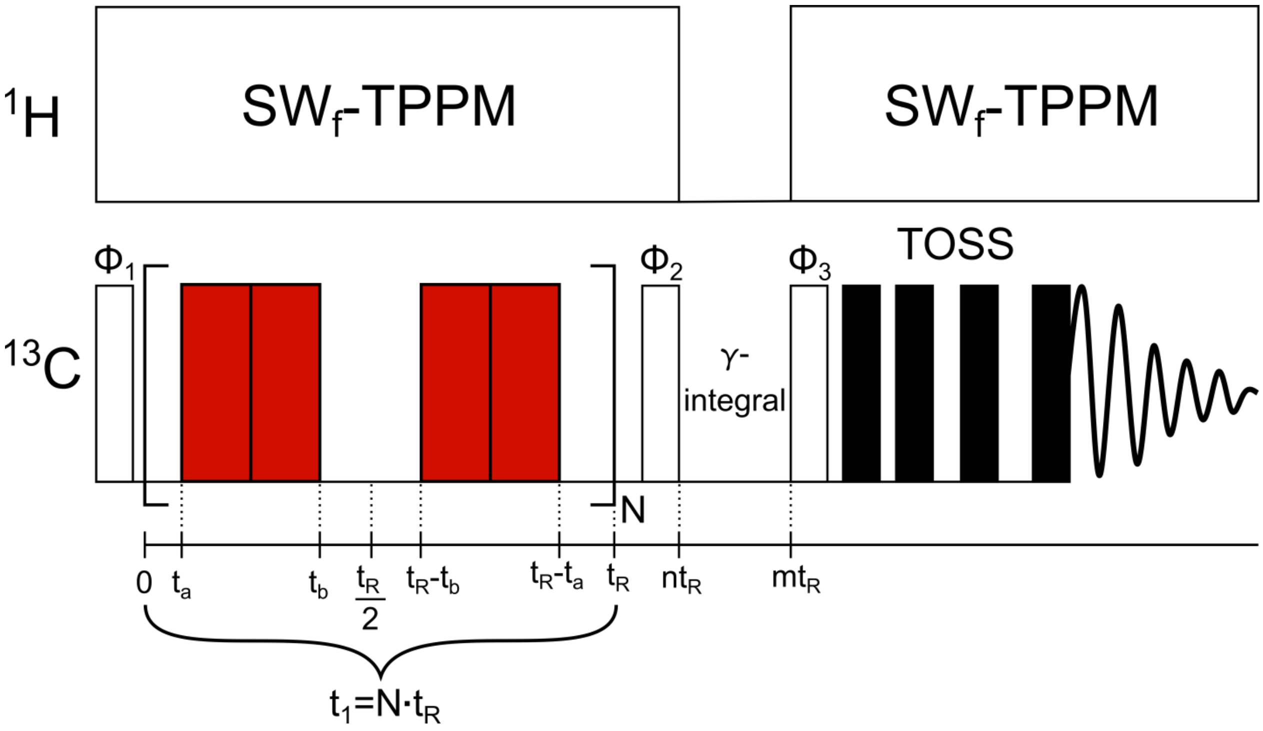

Fig. S3. Pulse sequence for the SUPER experiment used in this work. The white, black and red rectangles represent 90°, 180° and 360° pulses respectively. The phase cycle in multiples of 90° was Φ1=13203102, Φ2=11223300 and Φ3=33001100. The 360° pulses were phase cycled according to the original sequence by Liu et al..[1] The receiver phase was Φrec=02132031.

SIMPSON script used to simulate the SUPER spectra

```
# starts with Ix polarization, no phasecycling

# works fine

# takes a few seconds


spinsys {

 channels 13C

 nuclei   13C

 shift 1 1.5p -120p 0.09 0 0 0

}


par {
```

```
    spin_rate        6000
    proton_frequency 400.15e6

    crystal_file     rep320
    gamma_angles     64
    sw               6000
    start_operator   I1x
    detect_operator  I1m
    conjugate_fid    false
    np               32
    variable p22     1e6/(12.12*spin_rate)/2
    variable rf      12.12*spin_rate
    variable d21     1e6*0.2464/spin_rate
    variable d22     0.5*1e6/spin_rate-4*p22-d21
}


proc pulseq {} {
    global par
    acq_block {
                    delay $par(d21)
                    pulse $par(p22) $par(rf) x
                    pulse $par(p22) $par(rf) x
                    pulse $par(p22) $par(rf) -x
                    pulse $par(p22) $par(rf) -x
                    delay $par(d22)
                    delay $par(d22)
```

```
                pulse $par(p22) $par(rf) -x
                pulse $par(p22) $par(rf) -x
                pulse $par(p22) $par(rf) x
                pulse $par(p22) $par(rf) x
                delay $par(d21)
                }
}

proc main {} {
global par
set f [fsimpson]
fzerofill $f 128
faddlb $f 200 1
fft $f
set spename [fsave $f $par(name).spe]

puts "Calc. time = [expr $par(tcalc)*1e-6] s"
funload $f
}
```

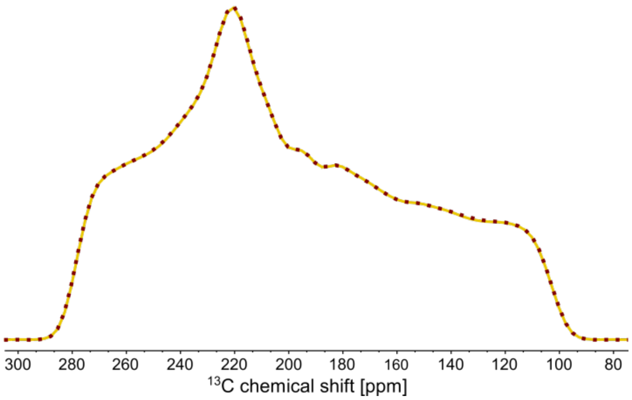

Fig. S4. Simulated line shape for a single (red, dotted) and two dipolar coupled spins (yellow) using SUPER and the following spin system: $\delta_1^{iso} = 1.5$ ppm, $\delta_1^{aniso} = 120$ ppm, $\eta_1 = 0.6$, $\delta_2^{iso} = -57.7$ ppm, $\delta_1^{aniso} = 80$ ppm, $\eta_1 = 0.6$ and $b_{12} = -386$ Hz. The Euler angles α, β and γ were always set to 0.

References

(1) Liu, S.-F.; Mao, J.-D.; Schmidt-Rohr, K. A Robust Technique for Two-Dimensional Separation of Undistorted Chemical-Shift Anisotropy Powder Patterns in Magic-Angle-Spinning NMR. *J. Magn. Reson.* **2002**, *155* (1), 15–28. https://doi.org/10.1006/jmre.2002.2503.
